# The association between erectile dysfunction and depression: a cross-sectional study of 21,139 Brazilian men

**DOI:** 10.31744/einstein_journal/2024AO1063

**Published:** 2024-11-22

**Authors:** Rafael Mathias Pitta, Oskar Kaufmann, Julio Silva Nogueira Luz, Raphael Mendes Ritti-Dias, Luana de Lima Queiroga, Nelson Wolosker

**Affiliations:** 1 Hospital Israelita Albert Einstein Faculdade Israelita de Ciências da Saúde Albert Einstein São Paulo SP Brazil Postgrad Program in Health Sciences, Faculdade Israelita de Ciências da Saúde Albert Einstein, Hospital Israelita Albert Einstein, São Paulo, SP, Brazil.; 2 Universidade Nove de Julho São Paulo SP Brazil Postgrad Program Rehabilitation Sciences, Universidade Nove de Julho, São Paulo, SP, Brazil.; 3 Universidade de São Paulo Faculdade de Medicina São Paulo SP Brazil Faculdade de Medicina, Universidade de São Paulo, São Paulo, SP, Brazil.; 4 Hospital Israelita Albert Einstein São Paulo SP Brazil Hospital Israelita Albert Einstein, São Paulo, SP, Brazil.

**Keywords:** Erectile dysfunction, Depression, Exercise, Life style, Obesity

## Abstract

**Objective:**

This study aimed to investigate the relationship between depression and erectile dysfunction while controlling for sociodemographic, physical health, psychological, and lifestyle factors. Additionally, it aimed to compare erectile dysfunction with other clinical, laboratory, and behavioral variables among individuals with depression in the same population.

**Methods:**

We included 21,139 men (≥40 years) who underwent continuous health screening in a quaternary hospital in São Paulo between January 2008 and December 2018. The analysis included laboratory, clinical, and behavioral profile data.

**Results:**

The multivariate model indicated that alcohol consumption, perceived stress, lower urinary tract symptoms, erectile dysfunction, body mass index, and triglyceride levels were independent and strong factors associated with depression in men. Additionally, higher levels of physical activity were found to be an independent and strong factor associated with reduced odds of depression in men (all p<0.001). Additionally, depressed men had a higher body mass index (28.50±4.35 *versus* 27.65±3.93kg/m², p=0.003), and increased prevalence of hypertension, *diabetes mellitus*, lower urinary tract symptoms, metabolic syndrome, nonalcoholic fatty liver, sedentary physical activity levels, active tobacco use, risk of alcohol consumption, and perceived stress.

**Conclusion:**

Erectile dysfunction was an independent and strong factor associated with depression in Brazilian men, highlighting the need for integrated treatment approaches addressing both conditions.

## In Brief

Pitta et al. demonstrated that moderate and severe lower urinary tract symptom were associated with higher odds of depression in 20,139 Brazilian men. Therefore, public authorities and private clinics should focus on actively promoting treatment and preventive measures for lower urinary tract symptoms to potentially reduce the risk of depression.

## Highlights

■ Levels of lower urinary tract symptoms increased the risk of depression.
■ Erectile dysfunction, alcohol consumption, and body mass index were associated with an increased likelihood of depression.

## Graphical abstract

**Figure:**
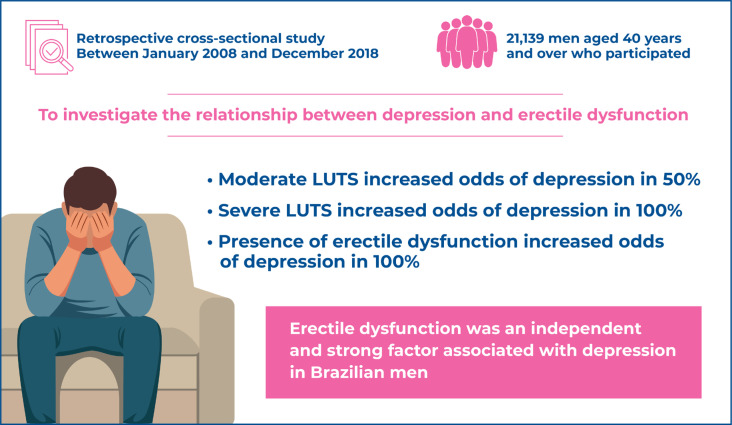

## INTRODUCTION

Erectile dysfunction (ED) is the persistent inability to have and/or maintain an erection sufficient for satisfactory sexual performance.^(1)^ The prevalence and incidence of ED in men increases with age, and it is estimated that approximately 322 million individuals worldwide will be affected by this condition within the next five years.^(2,3)^ Additionally, ED is associated with various issues, including ejaculatory disorders,^(4)^ cardiovascular diseases,^(4)^ lower urinary tract infections,^(4)^ and reduced quality of life for both men and their partners, owing to its effects on both physical and psychosocial health. It is also considered a risk factor for depression.^(2,4)^ However, the causal relationship between ED and depression is controversial; depression may increase the risk of developing ED, while ED may also worsen depression levels.^(5)^

Several studies have examined the association between depression and ED. However, few cross-sectional studies have explored whether ED is a risk factor for depression.^(5–7)^ Chou et al.^(5)^ conducted a 5-year study involving 15,162 Taiwanese patients to investigate the association between ED and depression. They defined ED using the International Classification of Diseases, Ninth Revision, Clinical Modification (ICD-9-CM), and depression was identified based on at least two depression diagnoses during ambulatory visits or at least one diagnosis during inpatient care. Their findings revealed that patients with ED had a 2.24-fold higher hazard ratio for developing depression over a 5-year follow-up compared to the comparison cohort, even after adjusting for age, urbanization level, geographic region, monthly income, and diseases.

Takao et al.^(6)^ investigated the relationship among lower urinary tract symptoms, ED, and depression in 87 Japanese patients with late-onset hypogonadism. Depression was assessed using the Mini International Neuropsychiatric Interview (MINI) questionnaire, and ED was evaluated using the short version of the International Index of Erectile Function. They found that younger age (<55 years) and moderate (OR=2.11, 95%CI=0.476-9.370) and severe ED (OR=2.99, 95%CI=0.959-9.332) were risk factors for depression.

Laumann et al.^(7)^ observed a relationship between lower urinary tract symptoms, depression, and ED in 2,173 Hispanic patients in the United States using data from the Male Attitudes Regarding Sexual Health study. They found that the odds of lower urinary tract symptoms were increased in depressed men (OR=2.68, 95%CI=1.60-4.47, p<0.01), as well as in men with ED (OR=1.73, 95%CI=1.11-2.71, p<0.05). Additionally, age was a factor, with men aged 60 to 69 years showing increased odds (OR=1.99, 95%CI=1.14-3.46, p<0.05) and those aged ≥70 years also exhibiting higher odds (OR=1.91, 95%CI=1.06-3.43, p<0.05). The odds of depressive symptoms were increased in men reporting lower urinary tract symptoms (OR=2.64, 95%CI=1.57-4.43, p<0.001), in men aged 50 to 59 years (OR=133.17, 95%CI=18.40-963.87, p<0.01), and in black men older than 60 years (OR=21.61, 95%CI=3.04-153.55, p<0.01).

While the relationship between ED and depression has been established, previous studies were based on small sample sizes. Furthermore, these studies often did not account for important confounding factors, such as clinical and behavioral factors, and did not use standardized questionnaires to assess depression and ED. This limitation restricts a comprehensive understanding of the multifactorial relationships between these variables. Additionally, cultural and sociodemographic factors can influence perceptions of depression and ED^(1–8)^ and affect how individuals seek treatment for these conditions. Understanding the epidemiology of depression and ED is essential for developing effective preventive strategies. Furthermore, to date, no Latin American publication has analyzed the relationship between ED and depression in a sample of over 10,000 patients using specific questionnaires.

## OBJECTIVE

This study aimed to investigate the relationship between depression and erectile dysfunction while controlling for sociodemographic, physical health, psychological, and lifestyle factors. Additionally, we aimed to compare erectile dysfunction and other clinical, laboratory, and behavioral variables among individuals with depression in the same population.

## METHODS

### Study design

In this retrospective cross-sectional study, we examined the link between depression and ED in Brazilian men aged 40 years and older. We included individuals who underwent health screening at the Preventive Medicine Center at a quaternary hospital in São Paulo between 2008 and 2018.

This study was approved by the Research Ethics Committee of *Hospital Israelita Albert Einstein* (CAAE: 94867018.6.0000.0071; # 2.844.247), and a waiver of informed consent was requested and granted.

### Participants and settings

Initially, data from 44,395 male health check-ups were included in this study. Men were excluded from the analysis under the following conditions: a) if they had undergone more than two health screenings, only the most recent visit was considered, and b) if there were duplicate or missing data for depressive symptoms and ED. Finally, data from 21,139 men aged 40 years and older were analyzed.

### Measurements

All individuals undergoing evaluation had their demographic, anthropometric, and clinical data collected. Trained doctors conducted all assessments, following strict protocols for data collection and storage.

Age was recorded as part of the preventive medicine assessment. Demographic assessment included weight (measured in kilograms) and height (measured in meters) using the InBody 230 (Ottoboni^®^) machine. The body mass index (BMI) was calculated using the weight/height^2^ formula (kg/m²).

Clinical data included blood pressure, comorbidities, laboratory results, and lifestyle factors. Blood pressure was measured three times, and the average of these measurements was used, following the guidelines of the American Heart Association^(9)^ after the patients had rested for at least 10 minutes. The measurements were conducted on both arms using the auscultatory method with an aneroid sphygmomanometer and phases I and V of the Korotkoff sounds. If the necessary data were unavailable in the medical assessment, the presence of hypertension and diabetes was based on self-reported information from the patients, including chronic use of antihypertensive or antidiabetic medication or self-reported *diabetes mellitus*.

Metabolic syndrome was defined in accordance with guidelines of the World Health Organization.^(10)^ Comorbidities such as systemic arterial hypertension, *diabetes mellitus*, dyslipidemia, tobacco use, nonalcoholic fatty liver steatosis (NASH), and continuous medication use were assessed based on medical records.

Laboratory data, including glycosylated hemoglobin percentage (HbA1C, %), standard lipid panel (mg/dL), creatinine (mg/dL), thyroid-stimulating hormone (TSH, mU/L), ultrasensitive C-reactive protein high (PCR, mg/dL), and total prostate-specific antigen (PSA, ng/mL), were collected after an overnight fast. These measurements were standardized based on the criteria established by the Brazilian Health Ministry and were conducted in the same laboratory.

Lifestyle factors were assessed using dedicated, validated questionnaires administered by trained professionals. Alcohol consumption was evaluated using the Alcohol Use Disorders Identification Test (AUDIT).^(11)^ Lower urinary tract symptoms (LUTS) were assessed using the International Prostate Symptom Score.^(12)^ Perceived stress was assessed using the Perceived Stress Scale (PSS-10).^(13)^ Physical activity levels (PAL) were measured using the International Physical Activity Questionnaire (IPAQ).^(14)^ ED was evaluated using a short version of the International Index of Erectile Function (IIEF-5).^(15)^

### Depression

The presence and severity of depression were evaluated using the Beck Depression Inventory (BDI-II).^(16)^ This questionnaire covers the past 15 days and contains 21 sets of statements about depressive symptoms. These statements are rated on a 0-3 ordinal scale, resulting in total scores ranging from 0 to 63. The severity of depression is classified as follows: a) 0-13 points indicate minimal or no depression, b) 14-19 points signify mild depression, c) 20-28 points indicate moderate depression, and d) 29-63 points signify severe depression. In this study, depression scores were categorized into two groups: presence (≥14 points, including mild, moderate, and severe depression) and absence (<14 points). This grouping was utilized for constructing logistic regression models and performing comparative tests.

### Data analyses

The normality of the data was assessed using the Shapiro-Wilk test. Categorical variables were analyzed using frequency and percentage distributions, while continuous variables were summarized using mean and standard deviation (SDs). General data were compared between the presence and absence of depression using the *t*-Student test for continuous variables and the X^2^ test for categorical variables. The association between depression and ED was tested using logistic regression models (using the backward stepwise method). The backward stepwise method is a regression approach that begins with a full model (Table 1S, Supplementary Material) and gradually eliminates variables at each step. This process continues until a reduced model is identified that best explains the data. For this analysis, depression scores were categorized as either present or absent. Statistical significance was set at p<0.05. Adjusted odds ratios (aOR) and 95% confidence intervals (95%CIs) were computed for logistic model results. Statistical analyses were performed using SPSS for Windows (version 24.0; IBM Corp., Armonk, NY, USA).

## RESULTS

We conducted a study on 21,139 men aged 40-91 years and found that 2,839 (13.43%) had depression. Table 1 presents a comparison of demographic and anthropometric data concerning depression. BMI was significantly higher in depressed men (28.50±4.35 *versus* 27.65±3.93kg/m², p=0.003).

**Table 1:** Comparison of demographic and anthropometric data in relation to depressive symptoms

Variable	Depressive symptoms	Mean	SD	n	p value
Age	Absent	49.96	7.50	18,242	<0.375
	Present	49.82	7.87	2,897	
	Total	49.94	7.55	21,139	
BMI (kg/m^2^)	Absent	27.65	3.93	18,240	0.003*
	Present	28.50	4.35	2,899	
	Total	27.77	4.00	21,139	

* *t*-student test.

BDI: beck depression inventory; SD: standard deviation; BMI: body mass index.

Depressed men had higher rates of hypertension, *diabetes mellitus*, LUTS, ED, metabolic syndrome, and NASH (Table 2; p<0.001 for all comparisons).

**Table 2:** Relative frequencies of comorbidities in relation to depressive symptoms

Variable		Depressive symptoms		Total n (%)	p value
		Absent n (%)	Present n (%)		
Hypertension		4,703 (25.70)	847 (29.80)	5,560 (26.30)	
*Diabetes mellitus*		1,445 (7.90)	307 (10.80)	1,754 (8.30)	
LUTS					<0.001*
	Absent	16,964 92.70)	2,453 (86.40)	19,427 (91.90)	
	Moderate	1,208 (6.60)	324 (11.40)	1,522 (7.20)	
	Severe	128 (0.70)	63 (2.20)	190 (0.90)	
Erectile dysfunction					<0.001*
	Absent	15,555 (85.00)	2,021 (71.20)	17,566 (83.10)	
	Presence	2,745 (15.00)	818 (28.80)	3,573 (16.09)	
	Dyslipidemia	9,882 (54.00)	1,465 (51.60)	11,347 (53.67)	
	Metabolic syndrome	1,867 (10.20)	395 (13.90)	2,262 (10.70)	
	NASH	9,296 (50.80)	1,666 (58.70)	10,962 (51.90)	

* χ^2^ test.

LUTS: lower urinary tract symptoms; NASH: nonalcoholic fatty liver steatosis.

Table 3 presents the results of the laboratory tests related to depression. High-density lipids were significantly higher in non-depressed men (45.68±11.14 *versus* 44.30±11.27 mg/dl, p<0.001), while triglyceride (TG) levels were significantly higher in depressed men (165.44±132.55 *versus* 148.16±108.01mg/dl, p<0.001). Additionally, depressed men had higher HbA1C (5.67±0.87 *versus* 5.60±0.73%, p<0.001), TSH (2.61±4.62 *versus* 2.42±2.18mU/L, p<0.001), ultrasensitive C-reactive protein (2.99±5.82 *versus* 2.55±5.69mg/dL, p=0.004), and creatinine (0.95±0.40 *versus* 0.94±0.16mg/dL, p=0.002) than had non-depressed men.

**Table 3:** Laboratory test results in relation to depressive symptoms

Variable	Depressive symptoms	Mean	SD	p value
TC (mg/dL)	Absent	194.69	38.73	<0.003*
	Present	186.34	38.35	
	Total	195.01	39.05	
HDL (mg/dL)	Absent	45.68	11.14	<0.001*
	Present	44.30	11.27	
	Total	45.49	11.17	
TG (mg/dL)	Absent	148.16	108.01	<0.001*
	Present	165.44	132.55	
	Total	150.53	111.852	
LDL (mg/dL)	Absent	120.91	34.07	0.230
	Present	121.74	35.30	
	Total	121.03	34.24	
HbA I c (%)	Absent	5.60	0.73	<0.001*
	Present	5.67	0.87	
	Total	5.61	0.75	
PSA total (ng/dL)	Absent	1.11	2.16	0.442
	Present	1.08	1.14	
	Total	1.11	2.04	
TSH (mU/L)	Absent	2.42	2.18	<0.001*
	Present	2.61	4.62	
	Total	2.45	2.66	
Ultrasensitive C-reactive protein high (mg/dL)	Absent	2.55	5.69	0.004*
	Present	2.99	5.82	
	Total	2.61	5.70	
Creatinine (mg/dL)	Absent	0.94	0.16	0.002*
	Present	0.95	0.40	
	Total	0.94	0.21	

* *t*-student test.

SD: standard deviation; TC: total cholesterol; HDL: high-density lipids; TG: triglycerides; LDL: low-density lipids; HbAIc: glycosylated hemoglobin; PSA: prostate-specific antigens; TSH: thyroid stimulating hormone.

Regarding lifestyle factors, men with depression were more sedentary and exhibited a higher prevalence of active tobacco use, risk of alcohol consumption, and perceived stress (p<0.001 for all; Table 4).

**Table 4:** Relative frequencies of modifiable lifestyle

Variable		Depressive symptoms		Total n (%)	p value
		Absent n (%)	Present n (%)		
IPAQ					<0.001*
	Sedentary	3,386 (18.50)	880 (31.00)	4,270 (20.20)	
	Low active	5,234 (28.60)	920 (32.40)	6,151 (29.10)	
	Active	7,448 (40.70)	869 (30.60)	8,308 (39.30)	
	Very active	2,232 (12.20)	170 (6.00)	2,409 (11.40)	
Tobacco use					<0.001*
	Never	12,975 (70.90)	1,877 (66.10)	14,861 (70.30)	
	Previous	3,678 (20.10)	661 (23.30)	4,333 (20.50)	
	Active	1,647 (9.00)	301 (10.60)	1,945 (9.20)	
Alcohol consumption					<0.001*
	Low-risk	15,555 (85.00)	2,090 (73.60)	17,630 (83.40)	
	Hazardous	2,489 (13.60)	579 (20.40)	3,065 (14.50)	
	Moderate-severe	256 (1.40)	170 (6.00)	444 (2.00)	
Perceiver stress					<0.001*
	Absent	15,994 (87.40)	920 (32.40)	16,806 (79.50)	
	Present	2,306 (12.60)	1,919 (67.60)	4,333 (20.50)	

* χ^2^ test.

IPAQ: International physical activity questionnaire.

Table 1S, Suplementar Material in the Supplementary Material presents the complete multiple logistic regression model. Following this, we conducted a stepwise backward multiple logistic regression, focusing on variables associated with either increased risk or protective factors for depression (Table 5). Physical activity levels were identified as strong independent protective factors against depression, with higher activity levels providing greater protection (p<0.001). In contrast, alcohol consumption (p<0.001), perceived stress (p<0.001), LUTS (p<0.001), ED (p<0.001), BMI (p<0.001), and TG (p=0.004) emerged as significant independent risk factors for depression.

**Table 5:** Predictors of depressive symptoms

Variable		OR	(95%CI)	p value
IPAQ				
	Low active	0.885	(0.778-1.007)	0.065
	Active	0.651	(0.572-0.741)	<0.001*
	Very active	0.512	(0.417-0.629)	<0.001*
Alcohol consumption				
	Hazardous	1.507	(1.325-1.714)	<0.001*
	Moderate-severe	3.474	(2.673-4.515)	<0.001*
	Perceived stress	13.398	(12.139-14.787)	<0.001*
LUTS				
	Moderate	1.505	(1.275-1.778)	<0.001*
	Severe	2.008	(1.345-3.000)	<0.001*
	Erectile dysfunction	2.021	(1.797-2.272)	<0.001*
BMI (kg/m^2^)		1.026	(1.014-1.038)	<0.001*
HDL (mg/dL)		0.995	(0.990-1.000)	0.047*
TG (mg/dL)		1.001	(1.000-1.001)	0.004*

* Logistic regression using the backward stepwise selection method.

OR: odds ratio; 95%CI: 95% confidence interval; IPAQ: International physical activity questionnaire; BMI: body mass index;kg/m^2^: kilogram/square meter; LUTS: Lower urinary tract symptoms; TG: triglycerides; HDL: high-density lipids.

## DISCUSSION

In this study, we provided a comprehensive assessment of the regional prevalence and predictive factors of depression in Brazil, using a validated questionnaire for evaluation. Our findings indicate that ED doubles the risk of depression, which is consistent with previous studies.^(2,17)^ Although the exact mechanism underlying this link is not fully understood, behavioral and biological models have been proposed to explain this causality. According to the behavioral model, depression may lead to negative thoughts and reduced self-confidence, resulting in performance anxiety, which further reduces erectile function.^(18)^ Additionally, individuals may experience reduced sexual desire, mood changes, and decreased responsiveness to sexual stimulation, all of which may contribute to ED.^(18)^

The biological model suggests that dysregulation of the hypothalamic-pituitary-adrenocortical axis, which is often observed in depressive symptoms, can lead to ED.^(19)^ This dysregulation can cause excessive catecholamine production, resulting in poor cavernosal muscle relaxation and ED.^(20)^ Furthermore, depression increases the risk of cardiovascular diseases and other comorbidities related to systemic inflammation, reduced penile oxygen flow, and ED.^(19)^ Our findings reinforce the importance of emotional wellbeing in ED prevention. It is important to encourage emotional balance as an effective approach for improving sexual function and cardiovascular health in men of all ages.

Alcohol consumption is a significant risk factor for depression, with hazardous and moderate-to-severe alcohol dependence increasing the likelihood of depression by 1.5 and 3.5 times, respectively. Heavy alcohol consumption can depress the central nervous system, leading to anxiety-like behavior and disruption of the brain's reward system.^(21)^ The strength of the relationship between alcohol use and depression varies depending on the measurement approach used. The link between depression and alcohol use was found to be strongest for heavy drinking and high quantity per occasion, less associated with volume, and unrelated to drinking frequency.^(21)^ This highlights the importance of the type of alcohol measurement and the need to assess various drinking levels to fully understand the alcohol-depression relationship. Lastly, our data demonstrate the emotional impact and alcohol dependence commonly observed among Brazilian executives facing high work demands and daily stress.

Similarly, perceived stress was associated with a 13-fold increase in the risk of depression. Stressful events, such as losing a job or a loved one, are known to contribute to the development of depression.^(20)^ These events trigger psychological and physiological changes, including the activation of the hypothalamic-pituitary-adrenal (HPA) axis and the sympathetic nervous system. This activation leads to alterations in the prefrontal cortex and affects the levels of glucocorticoids, glutamate, vasopressin, serotonin, and brain-derived neurotrophic factors, all of which are recognized as psychological stress responses.^(22)^

We observed that the presence and severity of LUTS were associated with depression. In a longitudinal study involving 9,080 adult Korean men, Rhee et al. found that LUTS were associated with a 1.8-fold higher hazard rate for developing depression after controlling for confounding factors. Emotions related to LUTS, such as fear, anxiety, worries about the future, embarrassment, helplessness, and loss of confidence, have been linked to the development of depression.^(22)^ Additionally, the presence and severity of LUTS are associated with ED,^(18)^ which is a significant risk factor for depression. Common biological pathways, including serotonin and norepinephrine levels, inflammation, phosphodiesterase isoenzymes, and the HPA axis,^(4)^ may underlie the association between LUTS and depression. Although storage symptoms are typically more bothersome than voiding symptoms, this study suggests that bothersome symptoms alone may not fully explain the longitudinal association with depressive symptoms.^(23)^

Our findings indicate that every 1kg/m² increase in weight raises the risk of depression by 2%. A previous longitudinal meta-analysis reported a larger combined effect size (ORs between 1.20 and 1.58).^(24)^ In this context, the passage of time may have a role in the association between depression and obesity. The negative impact of depression on the onset of obesity as well as the effect of obesity on the onset of depression may intensify over time.^(24)^ Obesity results from various lifestyle changes, such as poor diet, increased anxiety and stress, increased alcohol consumption, worsened sleep, and a sedentary lifestyle. It creates an inflammatory state, as weight gain has been shown to activate inflammatory pathways. Given the involvement of inflammation in both obesity and depression, it is possible that inflammation serves as the mediator of the association.^(25)^ Furthermore, it is widely recognized that dysregulation of the HPA axis is implicated in depression. Such dysregulation may lead to the development of depression as a consequence of obesity.^(26)^

Research indicates that physical activity is linked to a lower risk of depression, even when controlling for other risk factors. Even small amounts of physical activity can reduce the risk of depression. A recent meta-analysis by Schuch et al.^(27)^ demonstrated that higher levels of physical activity are associated with lower odds of developing depression compared to a sedentary lifestyle, even after adjustments were made (adjusted odds ratio=0.83, 95%CI=0.79-0.88, p=0.001; I²=0.00). However, it is important to note that there may be some bias in the research findings. Dishman et al. also noted a dose-response association, using meta-regression, between PAL and depression (adjusted odds ratio 0.79, 95%CI=0.75-0.82; p=0.001, I^2^=87.6). This association can be explained by acute neuroendocrine and inflammatory responses to physical activity, such as activation of the endocannabinoid system.^(28)^ Furthermore, long-term adaptations, including changes in the brain's neural architecture, may be observed.^(29)^ Physical activity levels improve physical self-perception, body image, social interactions, and the personal development of coping strategies. The social aspect of activity participation may operate even at relatively low doses, consistent with the dose-response curve, and should be encouraged in this population to prevent depressive symptoms.

Our study has some limitations and several strengths. First, this study followed a cross-sectional design, which prevents us from establishing a causal relationship between ED and depression. Second, we used self-reported questionnaires to measure depression and other lifestyle variables. Third, our study excluded men aged <40 years and only included men with private insurance who participated in health check-ups, raising the risk of selection bias, which may limit the generalizability of our findings to the entire Brazilian male population. The strengths of this study include its thorough analysis of depression in Brazil. All participants underwent a detailed, complete, and validated health examination by a physician, including an assessment of the prevalence and severity of depression, ED, and behavioral levels. This reinforces the reliability and validity of the results. Additionally, we used a logistic regression model with clinical, anthropometric, demographic, and behavioral factors to provide a comprehensive understanding of depression in men. We anticipate that our study may serve as a preventive tool in clinical and public health settings, guiding the management of depressive symptoms and other emotional disorders associated with ED. While our study did not establish a causal relationship, it may inform depression prevention strategies and encourage lifestyle improvements among men. We also recommend conducting longitudinal studies to further explore the impact of changes in these outcomes within this population and in other groups.

## CONCLUSION

In conclusion, erectile dysfunction is associated with a higher risk of depression. Furthermore, various clinical factors (lower urinary tract symptoms, body mass index, and triglyceride levels) and behavioral factors (perceived stress and alcohol consumption) are independent factors associated with depression in the same population. Additionally, higher levels of physical activity are associated with reduced odds of experiencing depression.

## SUPPLEMENTARY MATERIAL The association between erectile dysfunction and depression: a cross-sectional study of 21,139 Brazilian men

Rafael Mathias Pitta, Oskar Kaufmann, Julio Silva Nogueira Luz, Raphael Mendes Ritti-Dias, Luana de Lima Queiroga, Nelson Wolosker

**DOI: 10.31744/einstein_journal/2024AO1063**

**Table 1S:** Predictors of depressive symptoms in men

Variable		OR	(95%CI)	p value
Hypertension		0.942	(0.837-1.061)	0.328
*Diabetes mellitus*		1.134	(0.951-1.352)	0.162
Dyslipidemia		0.908	(0.816-1.010)	0.075
Metabolic syndrome		0.995	(0.837-1.182)	0.955
Tobacco				
	Never	1.046	(0.928-1.179)	0.464
	Previous	0.955	(0.804-1.134)	0.596
IPAQ				
	Low active	0.889	(0.781-1.012)	0.076
	Active	0.654	(0.574-0.745)	<0.001*
	Very Active	0.517	(0.420-0.635)	<0.001*
	NASH	1.036	(0.925-1.159)	0.545
Alcohol consmption				
	Hazardous	1.506	(1.323-1.715)	<0.001*
	Moderate-to-severe	3.481	(2.674-4.532)	<0.001*
	Perceiver stress	13.391	(12.132-14.781)	<0.001*
LUTS				
	Modetate	1.502	(1.271-1.774)	<0.001*
	Severe	2.004	(1.340-2.998)	0.001*
	Erectile dysfuntion	2.014	(1.790-2.267)	<0.001*
BMI (kg/m^2^)		1.024	(1.011-1.038)	<0.001*
HDL (mg/dL)		0.995	(0.990-1.000)	0.05
TG (mg/dL)		1.001	(1.000-1.001)	0.004*

* Full multiple logistic regression.

OR: odds ratio; 95%CI: 95%confidence interval; IPAQ: International physical activity questionnaire; BMI: body mass index;kg/m²: kilogram/square meter; LUTS: lower urinary tract symptoms; TG: triglycerides; HDL: high-density lipids.
